# Exploration of *Klebsiella aerogenes* derived secondary metabolites and their antibacterial activities against multidrug-resistant bacteria

**DOI:** 10.1371/journal.pone.0300979

**Published:** 2024-09-16

**Authors:** Syed Hussain Shah, Hsien Liu, Muddasir Khan, Riaz Muhammad, Abdul Qadeer, Dalia Fouad, Chien-Chin Chen

**Affiliations:** 1 Department of Health and Biological Sciences, Abasyn University Peshawar, Peshawar, Pakistan; 2 Division of General Surgery, Department of Surgery, Ditmanson Medical Foundation, Chia-yi Christian Hospital, Chiayi, Taiwan; 3 Centre of Biotechnology and Microbiology, University of Peshawar, Peshawar, Pakistan; 4 Department of Cell Biology, School of Life Sciences, Central South University, Changsha, China; 5 Department of Zoology, College of Science, King Saud University, Riyadh, Saudi Arabia; 6 Department of Pathology, Ditmanson Medical Foundation, Chia-Yi Christian Hospital, Chiayi, Taiwan; 7 Department of Cosmetic Science, Chia Nan University of Pharmacy and Science, Tainan, Taiwan; 8 Department of Biotechnology and Bioindustry Sciences, National Cheng Kung University, Tainan, Taiwan; 9 Ph.D. Program in Translational Medicine and Rong Hsing Research Center for Translational Medicine, National Chung Hsing University, Taichung, Taiwan; University of Buea, CAMEROON

## Abstract

As the effectiveness of current treatments against the development of antimicrobial resistance is declining, new strategies are required. A great source of novel secondary metabolites with therapeutics effects are the endophytic bacteria present in medicinal plants. In this study, *Klebsiella aerogenes* (an endophytic bacteria belonging to the *Enterobacteriaceae* family) was isolated from *Kalanchoe blossfeldiana* (a medicinal plant”. The bacterial secondary metabolites were identified using GC-MS techniques. Furthermore, the antibacterial potentials were investigated against multi-drug resistance (MDR) *Salmonella typhi* and *Staphylococcus aureus*. The GC-MS chromatogram of *K*. *aerogenes* secondary metabolites extract displayed total of 36 compounds. Ethyl acetate extracts of *K*. *aerogenes*, showed mean zone of growth inhibition of 15.00 ± 1.00 against *S*. *typhi* and 7.00 ± 1.00mm against *S*. *aureus*, respectively. The extract demonstrated significant antibacterial effectiveness against *S*. *typhi* and moderate antibacterial efficacy against *S*. *aureus*, with minimum inhibitory concentration (MIC) values ranging from 0.089 to 0.39 mg/mL. The time-kill kinetics profile of the ethyl acetate extract against *S*. *typhi* revealed a decrease in the number of viable cells during the initial 5, 6, and 24 hours. Conversely, there was a sudden increase in viable cells up to 6 hours for *S*. *aureus*. The identified secondary metabolite with high percentage than others, benzeneethanamine exhibited favorable interactions (−7.2 kcal/mol) with the penicillin-binding protein (PBP2a) of *S*. *aureus* and (−7.5 kcal/mol) osmoporin (OmpC) of *S*. *typhi*, indicating its potential as a candidate for drug development against these MDR bacteria. This study reported for the first time, bacterial endophytes associated with *K*. *blossfeldiana* with antibacterial activities.

## Introduction

Multi drug resistance (MDR) *Staphylococcus aureus*, a common gram-positive pathogen, leads to diverse infections with substantial morbidity and mortality in healthcare and community settings [1]. *S*. *aureus* holds the classification of a World Health Organization (WHO) Priority II pathogen due to its proclivity to induce both acute and chronic infections, coupled with its capacity to exhibit resistance to antibiotics. Its tremendous effect on healthcare systems worldwide is largely attributed to this dual characteristic [2]. Even after ten years of researching ways to fight infections caused by MDR S. *aureus*, it’s still a big problem [3]. MDR *S*. *typhi*, which is a type of gram-negative bacteria, specifically leads to typhoid fever in humans. This sets it apart from other types of *Salmonella* [4]. Typhoid fever burdens Africa, South Asia, and Southeast Asia with millions of cases and thousands of deaths. Pathogen persistence involves virulence plasmids and *Salmonella* pathogenicity islands [5]. In response to the urgent need for new antibiotics against MDR bacteria, the exploration of cave microorganisms for antimicrobial compounds has gained momentum [6].

Natural products (NPs) play pivotal roles in industry, biotechnology, and medicine, combating diseases like cancer and bacterial infections, shaping our society [7]. NPs screening reveals structurally unique, potent, and selective molecules sourced from fungi, plants, and bacteria [8]. Bacteria are a valuable source of bioactive secondary metabolites (SMs), greatly contributing to human medical well-being [9]. Extensive research has revealed specific bacterial SMs e.g. prodigiosin, doxorubicin, ixabepilone, chartreusin, elsamicins, monensin, etc. with potent antimicrobial properties against pathogenic strains, providing valuable insights for combating infectious diseases. Endophytic bacteria exhibit diverse metabolic traits, fostering plant growth, combating pathogens, and aiding in environmental adaptation [9, 10].

Endophytes yield abundant, unique, and biologically active SMs, benefiting both host plants and offering economic potential in pharmaceutical, agricultural and food sectors [11]. Recognizing the significance of endophytes in plant metabolites, recent investigations have unveiled the pivotal role of endophytes in medicinal plants [12]. Endophytic microbes offer diverse therapeutic SMs such as phenols, carboxylic acids, and proteins for drug discovery, holding promise in combating human pathogens and MDR strains [13]. Endophytes synthesize abundant bioactive SMs, benefiting host plants and exerting a vital impact on the pharmaceutical, agricultural, and food sectors [14].

The emergence of MDR bacteria poses a serious threat to world health, necessitating creative strategies to address antibiotic resistance. Therefore, the goal of this study was to explore untapped microbial diversity by isolating and identifying endophytic *Enterobacteriaceae* from *Kalanchoe blossfeldiana*, which may result in the discovery of new bioactive substances in order to effectively battle against MDR bacteria.

## Materials and methods

### Collection of the plant materials

The healthy fresh leaf samples from a total 50 number of *K*. *blossfeldiana* plants were collected at District Charsadda, Tehsil Shabqadar Forte (34°23’92.4” N, 71°57’25.2” E) in Khyber Pakhtunkhwa province of Pakistan. The leaves were carefully placed in sterile polyethylene bags and transported to the laboratory while maintaining a temperature of 4°C. The identification process for the leaves was conducted at Abasyn University in Peshawar, Pakistan. A specimen of the plant material was identified and officially stored in the botany department at Abasyn University in Peshawar, assigned the voucher specimen number K.025, and categorized under the species name *K*. *blossfeldiana*. Subsequently, the leaves were promptly processed in the laboratory.

### Isolation of bacterial endophytes

Bacterial endophytes were extracted from fresh leaves, approximately 3–5 leaves, obtained from a single entire plant. The isolation process adhered to the method developed by Ding and Melcher [15]. After obtaining pure colonies of bacterial endophytes, 35% glycerol stock cultures were prepared by diluting glycerol in sterile distilled water. These cultures were then stored at -80°C for future utilization. Stock cultures of a single bacterial isolate were retrieved from long-term storage and transferred onto fresh nutrient agar (NA) media. The cultures were then incubated for 2–7 days at 30°C. Sub-culturing of the chosen isolate was repeated multiple times until pure colonies were consistently obtained [16].

### Isolation and identification of *K*. *aerogenes*

The isolated bacteria were identified by colony morphology, and gram staining. Analytical Profile Index 20E (API) strip was used for biochemically identification of the *Enterobacteriaceae*. In brief, bacterial suspensions were rehydrated in the wells of strip, followed by incubation. Metabolism led to color changes, either spontaneous or with reagents. Positive and negative test results formed a profile number, compared with a commercial codebook for bacterial species identification was obtained [17]. Molecular identification of 16S rRNA was analyzed at Macrogen 238, Teheran-ro, Gangnam-gu, Seoul, Republic of Korea. ZR Bacterial Kit™ was used for RNA extraction in accordance with the manufacturer’s instructions. Following by BLAST the purified strain was analyzed using Bio Main Workbench CLC v7.6, utilizing the National Center for Biotechnology Information (NCBI) database search engine for classifying nearby bacterial species according to the method of [17].

### Extraction of secondary metabolites (SMs) from *K*. *aerogenes*

The endophytic bacteria were isolated from *K*. *blossfeldiana* and incubated in 1 liter of Luria Bertani (LB) broth and stirred at 200 rpm at 28°C for seven days. Following culture, the flask was further shaken at 180 rpm for two hours and 20 g/L of the Amberlite® XAD7HP 20–60 mesh (Sigma-Aldrich, Darmstadt, Germany) was added to absorb the secondary metabolites. The resin was filtered through a cheese cloth and then extracted three times with 200 mL of acetone. Using a rotary vapor concentrator (Lab Tech, Nantong, Jiangsu, China), the acetone was concentrated at 5°C until a viscous, dark brown extract was produced. A measuring cylinder was filled with the leftover water that contained the crude extracts, and the same volume of ethyl acetate (1:1 [v/v]) was then added. After agitating the liquid vigorously for five to ten minutes, a funnel was used to separate it. After three additional rounds of this procedure, the ethyl acetate fraction was vaporized by using a rotary vaporizer. After being placed into sterile beakers and sealed with foil, the crude extracts were allowed to dry at room temperature [16, 18].

### Analysis using gas chromatography-mass spectrometry (GC-MS)

The extract was analyzed using GC-MS analysis. GC oven was set for 3 min at 40°C initially and increased subsequently per min by 5 to 220°C. The helium gas flow rate was programmed at 1.0 mL/min with a split ratio of 10:1, and the injector temperature was set at 250°C. The MS system’s ion source was set to 250°C in temperature, and a voltage of 70 eV was supplied. The Rt-Q-Bond capillary column 30 m × 0.25 mm × 8 μm (Restek, Bellefonte, PA, United States) was used for analysis. The process of identifying and matching peaks, aligning peak and retention times, and detection will be carried out by NIST-17 library using Chroma TOF-HRT® software by LECO Corporation. Using a collection rate of 10 spectra per second, the mass fragments were employed ranged from 40–660 m/z. The ChromaTOF programme (LECO Corporation, St. Joseph, MI, USA) was used to analyze and interpret GC-MS mass spectra [19].

### Antibacterial activity

#### Preparation of test sample

The test sample was prepared by the method described by [20], to determine the endophyte crude extract antibacterial activity after making a few minor adjustments. The pure culture of isolated *K*. *aerogenes* was pre-cultured for 24 hours at 37°C. A loopful of fresh culture was inoculated to 50 mL sterilized NB in a 100 mL Erlenmeyer flask and placed for incubation for 72 h at 28°C, and 130rpm [20]. A 2 mL fermented broth was centrifuged for 15 minutes at 13,000 rpm. For antibacterial screening, the culture’s supernatant was used.

#### Test pathogens

The test pathogens; MDR *S*. *aureus* and *S*. *typhi* were kindly provided by the Centre of Biotechnology and Microbiology, University of Peshawar. The bacterial culture was inoculated in a sterile broth with the bacterial strains and the culture was incubated. The absorbance was measured at 600 nm using a UV-spectrophotometer Shimadzu. The test bacterial inoculum was adjusted to 106 CFU/mL by measuring the absorbance at 600nm wavelength [21].

#### Determination of minimum inhibition zone (MIZ)

Using sterile swabs, the test bacteria MDR S. *aureus a*nd S. *typhi* were streaked on the surface of nutrient agar (NA) plates. The bacterial lawn was divided into wells with a diameter of around 6 mm using a sterile cork borer. About 50μl supernatants of the centrifuged endophyte broth were pipetted to the well. 20μg/mL ciprofloxacin was set as the positive control, while nutrient broth set as the negative control. For the diffusion of the supernatant on the agar, the plates were left for 10min at room temperature inside the laminar flow hood. The plates were incubated at 37°C for 24h, and the minimum inhibition zone was observed. The inhibition zone was expressed and measured in millimeters, as indicated by antibacterial activity [17].

#### Determination of minimum inhibitory concentration (MIC)

The MIC was determined by the method described by [17] with slight modifications. About, 100μl of the freshly prepared test bacteria adjusted to McFarland 0.5 standard, was pipetted into a 10 mL test tube containing 1% dimethyl sulfoxide (DMSO) solvent. The test sample was tested in various concentrations. For negative control, 1% DMSO was utilized, while for positive control 20μg/mL ciprofloxacin hydrochloride working standard (Unichem Pharmaceuticals Pakistan) was used. After sealing, the tubes were kept overnight at 37°C. After incubation, the tube was observed by using UV-Spectrophotometry Shimadzu 1800 at 600m wavelength according to the protocols of [17]. The MIC is the lowest concentration of the extract that stops bacteria from growing [17].

#### Determination of minimum bactericidal concentration (MBC)

MBC was measured by pipetting 100μl from each tube that had no bacterial growth [22]. A sterile spreader was used to spread on different NA plates. The plates were kept in a 37°C incubator for 24 hours. Extract at the lowermost concentration that exhibits complete killing of the isolates was considered the MBC [17].

#### Determination of time kills kinetics

The concentration of the extract was made at one-half, two-fold, and four times it’s MIC. The 1.0⨯106 CFU/mL inoculum was added at 37°C and incubated. At different time intervals, i.e., zero, one, two, three, four, five, six, twelve, and twenty-four-hours. Aliquots of 1.0 mL of the medium were taken and aseptically inoculated into 20 mL NA and was incubated at 37°C for twenty-four hours. Without the reference standard antibiotic and extract a control test was performed. The organism CFU was determined. The log CFU/mL graph was plotted against time using the approach of [23], and all processes were examined in triplicate.

#### Computational analysis of protein-ligand interactions via molecular docking

Endophytic *K*. *aerogenes* antibacterial SMs were selected for molecular docking. The 3D structures of the target proteins responsible for cell wall synthesis penicillin binding protein (PBP2a) in MDR *S*. *aureus* (PDB ID = 5M18) and for MDR S. *typhi* osmoporin (OmpC) outer membrane protein (ID = 3UU2) was downloaded from the protein databank (http://www.rscb.org/pdb, accessed on 25 June 2023). The 3D structure of the selected ligand was downloaded from the PubChem database (www.pubchem.com) accessed in.sdf formats. The.sdf files of the selected files were converted into PDB formats by using Discovery Studio Software.

Following the method of [24] the protein was treated by using the Discovery Studio visualizer (https://discover.3ds.com/discovery-studio-visualizer-download). The water molecules and ligands were removed, purified, and saved in PDB format. The 3D structure of the selected ligand in the structure data file (SDF) format was first converted into PDB format in the Discovery Studio visualizer and then treated and purified by removing water molecules in the Discovery Studio visualizer. The purified ligand was saved in PDB format for further analysis.

The active site within the protein was identified using Auto Dock tools (https://autodock.scripps.edu/). Using Auto Dock tools, the polar hydrogen was also added to the PDB ID = 5M18 and (ID = 3UU2). The protein was initialized as a macromolecule and saved in PDBQT format. After that the grid box was also set, and the file was saved in grid.txt format. The ligand was prepared in the auto dock tools. By default, the torsion angle of 6 was added to the ligand and saved in PDBQT format. The file was manually prepared by adding the receptor and ligand pdbqt formats, out (out.pdqt), center, size, and exhaustiveness. The file was saved in confg.txt format. The command prompt was entered in the search box and the Vina folder path was selected. The config.txt, and log.txt files were entered in the command form and the docking result was initialized.

### Statistical analysis

All experiments, unless specified differently, were done in triplicate. Version 2010 of Microsoft Excel was used to compute the mean values. The significance of the variation in mean values was assessed using the t-test. The variance was calculated using a one-way ANOVA with p ≤ 0.05 significant values [16, 25].

## Results

### Isolation of endophytic *K*. *aerogenes*

**Fig 1:**
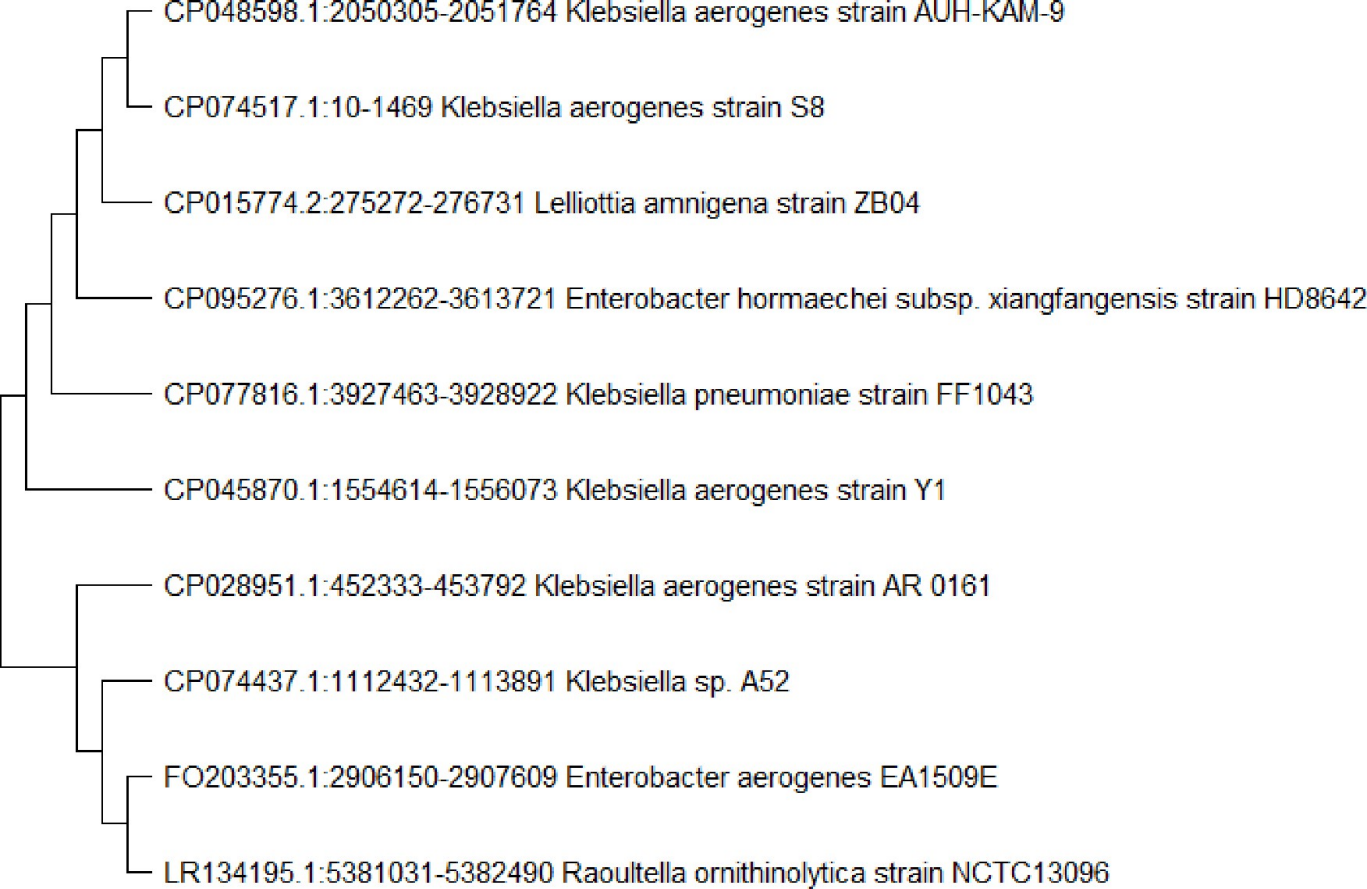
The isolated *K*. *aerogenes* phylogenetic tree by neighbor-joining method (Bootstrap analysis with 500 replicates) generated by MEGA-X software.

*K*. *blossfeldiana* plant leaves were immersed in 70% ethanol, 2% sodium hypochlorite, and 10% sodium bicarbonate in order to sterilise their surface. This protocol was effective, as the control plates did not reveal any bacterial growth after 5 days as shown in (**S1 Fig**). The selected *K*. *aerogenes* was identified as rod shape colony, gram-negative rod shape bacteria by gram staining when observed under a light microscope at 100X magnification using a compound bright-field microscope. The biochemical characterization of *K*. *aerogenes* was performed by Analytical Profile Index (API 20E) strip with catalase-positive, and oxidase-negative. The code was generated from the API 20E strip (53 057 73). The bacteria were identified as *K*. *aerogenes* when compared with the profile numbers in a commercial codebook. The 16S rRNA gene sequences (**S2 Fig**) study of *K*. *aerogenes* using NCBI database representative bacterial strains of related species **(Fig 1)**.

### GC-MS analysis of *K*. *aerogenes* extracts

SMs were extracted using a 1000 mL extraction flask. Ethyl acetate was used as a solvent. After evaporation of ethyl acetate, the isolate *K*. *aerogenes* yielded 0.6g/500 mL crude extract. GC-MS chromatogram displayed a total of 36 compounds, as shown in (**Table 1**), and (**S3 Fig**).

**Table 1:** Identified active constituents of ethyl acetate extract of *K*. *aerogenes* by GC-MS.

S. no.	Name of Compounds	R. Time	Area	Conc. (%)
1	3-methyl butanoic acid	3.263	3078721	8.26
2	5-dichloro 2,6-Lutidine-4-[benzyloxy]-3	3.928	1241671	2.64
3	3-(methylthio)1-propaneamine	4.180	1117589	4.03
4	Butoxyacetic acid	4.686	3458910	3.55
5	3-Hydroxy-2-methylthio-3-phenylpropanoic acid	5.179	1743828	7.47
6	1-Deoxy-d-arabitol	5.675	1117539	3.21
7	Benzeneethanamine	5.986	1245263	20.18
8	(S)-2-butanamine	6.178	1031921	4.38
9	2-amino-5-methyl-thiazole	6.473	392875	1.64
10	5-bis(1,1-dimethylethoxy)-thiophene	7.204	935547	6.95
11	2-bis(3,3-dimethyl-2oxobutylthio) propanedinitrile	8.074	1107168	8.45
12	Hydroxycarbamic acid ether ester	8.686	1801821	2.87
13	3-Aminopiperidin-2-one	9.016	4151664	3.07
14	1-(1-Cyclopenten-1-yl)-pyrrolidine	9.765	93287	3.28
15	dl-Cystathionine	10.143	572397	1.40
16	2-(diethylamino)- N-oxide Ethanol	10.989	593655	3.23
17	(E,E)-2,4-Heptadien-6-yn-1-ol	11.034	7738904	4.26
18	1,3-dipropyl-8-[4-[β-[(benzyloxycarbonyla xanthine	11.697	700244	9.81
19	16-octadecadiynoiate methyl	12.086	1299666	2.62
20	2-(2,5-Hexadiynyloxy) tetrahydro-2H-pyran	12.674	3160884	2.64
21	N-(p-methoxybenzyl) acetamide	12.968	1073523	1.47
22	2,4-Dimethyl hexanedioic acid	13.890	8843821	1.65
23	Oleic acid	14.060	1187392	1.83
24	Didodecyl phthalate	14.656	5823731	0.76
25	Methyl cyclopentane undecanoiate	15.148	7756901	1.64
26	2-Aminoethanethiol hydrogen sulfate	16.456	2678012	2.01
27	1-ethyl-isoquinoline	17.896	1214738	1.78
28	N-Benzyloxycarbonyl-dl-norleucine	18.578	583756	0.85
29	2-Acetyl-5-chloromethyl-isoxazolidin-3-one	19.787	4151766	6.57
30	2,5-Dimethyl-3-n-pentylpyrazine	19.987	592644	0.39
31	3-methyl-6-(1-methylethyl)-2,5-Piperazinedione	20.018	3270821	4.26
32	2,6-Dimethyl-3-sec-butylpyrazine	22.398	883446	1.34
33	3-Methyl-1,4-diazabicyclo[4.3.0]nonan-2,5-dione	23.645	916614	1.06
34	3-Pyrrolidin-2-yl-propionic acid	26.694	38387	0.65
35	2-phenylethyl ester cyclohexanecarboxylic acid	27.463	478433	3.04
36	N-formyl-tyramine	28.983	763890	2.53

### Antimicrobial properties of the extract

**Fig 2:**
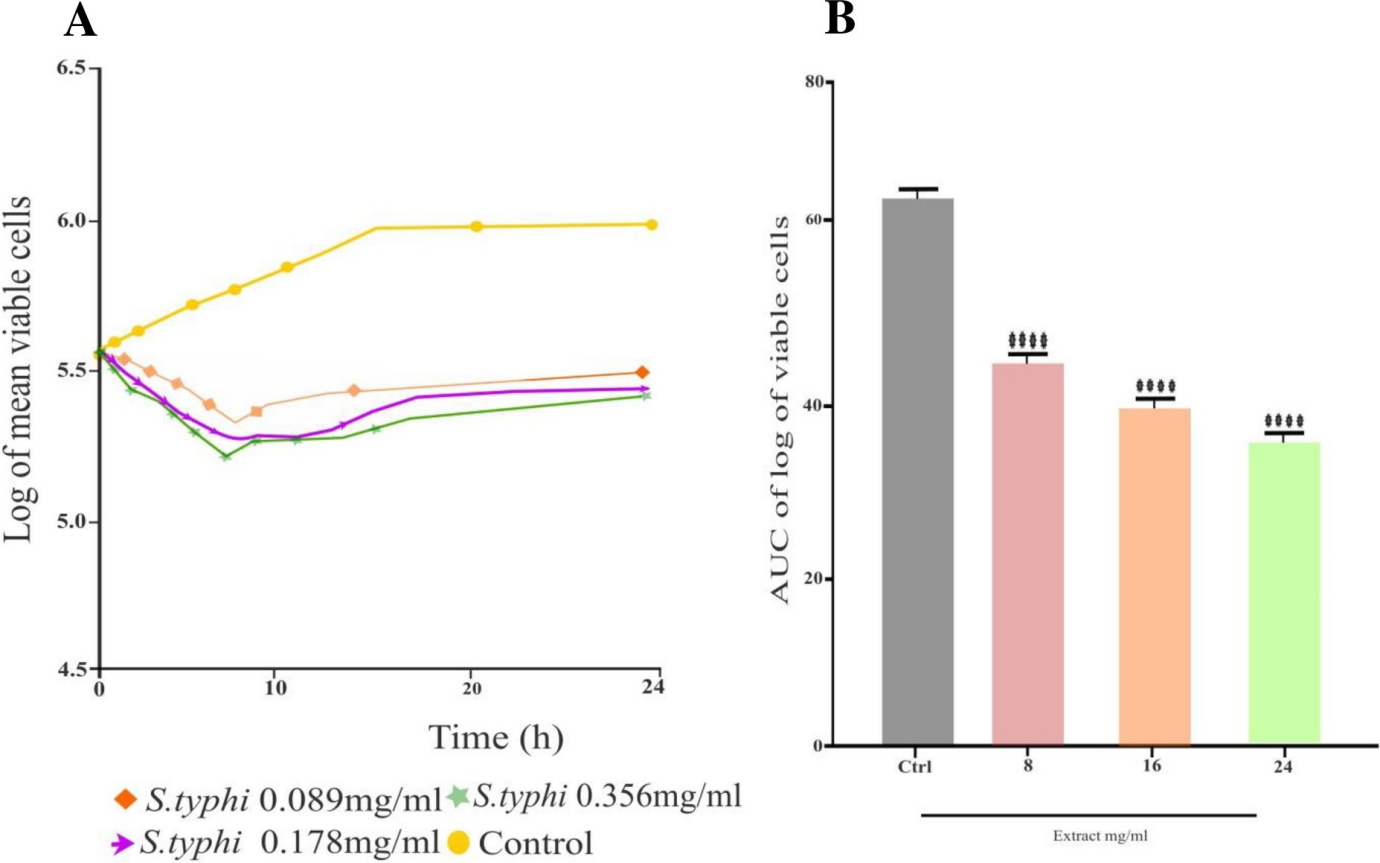
**A)** Time-kill kinetics curve of *S*. *typhi*, **B)** AUC of time kill kinetic of *S*. *typhi*.

**Fig 3:**
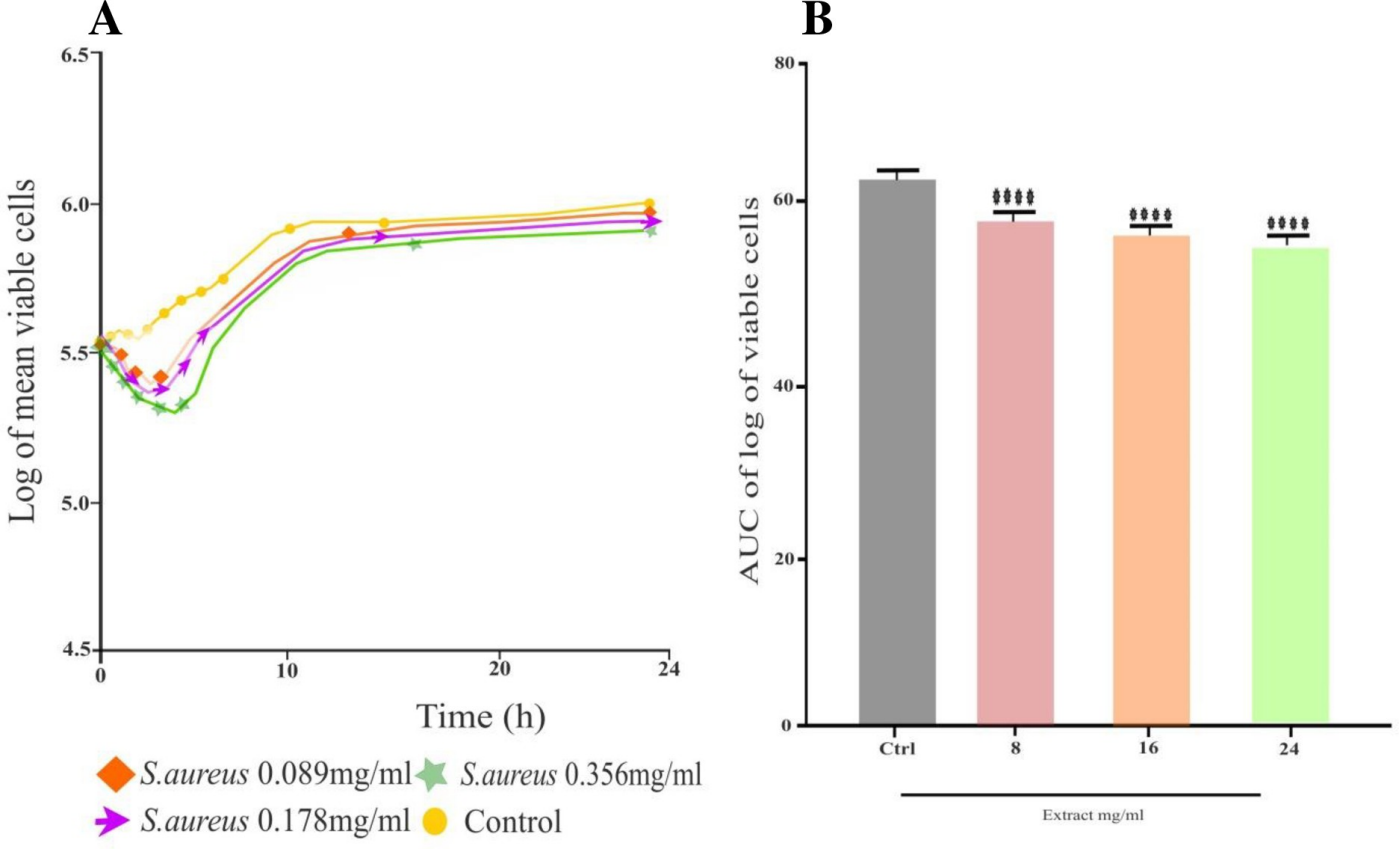
**A)** Time-kill kinetics curve of *S*. *aureus*, **B)** AUC of time kill kinetics of *S*. *aureus*.

The selected bacterial pathogens were utilized in the tests to determine the *K*. *aerogenes* extract antibacterial effectiveness. The extract demonstrated significant (p < 0.05) inhibitory effect using the agar diffusion method against MDR *S*. *typhi a*nd MDR *S*. *aureus*. The MIZ of 15 ± 1.00 mm indicated that it was mostly efficient against *S*. *typhi*, while there were very minute 7.00 ± 1.00mm significant inhibitions shown against *S*. *aureus*, as shown in (**Table 2**). The extract MIC varied from 0.089 mg/mL to 0.39 mg/mL. The extract was more profound against MDR *S*. *typhi* with a MIC of 0.39 mg/mL. However, it did not demonstrate remarkable antibacterial activity against MDR *S*. *aureus* (**Table 3**). The bactericidal effect (MBC) of the extract against *S*. *typhi* was found at 0.39mg/mL, while *S*. *aureus* did not show any bactericidal effect, as shown in (**Table 3**). When comparing *S*. *typhi* to extract test concentrations, the time-kill kinetics profile revealed a decrease in the number of viable cells over the first 5, 6, and 24 h as shown in (**Fig 2**) while rising up to 7 h against *S*. *aureus* (**Fig 3**).

**Table 2:** *K*. *aerogenes* extract antibacterial activity assessed via agar diffusion method.

Concentration	Zone of inhibition (mm)	
	MDR *S*. *typhi*	MDR *S*. *aureus*
50μl	15 ± 1.00mm	07 ± 1.25mm

**Table 3:** MIC and MBC values of the *K*. *aerogenes* extract against MDR strains.

	*S*. *typhi*		*S*. *aureus*	
Concentration of extracts	MIC (abs.)	MBC (CFU/mL)	MIC (abs.)	MBC (CFU/mL)
0.089mg/mL	0.753	189	0.813	500
0.152mg/mL	0.725	143	0.808	480
0.180mg/mL	0.707	105	0.803	350
0.390mg/mL	0.512	58	0.791	250
Positive control	0.028	No growth	0.038	No growth
Negative control	0.817	Numerous	0.881	Numerous

### Molecular docking analysis against the cell wall synthesis protein of the tested MDR strain

**Fig 4:**
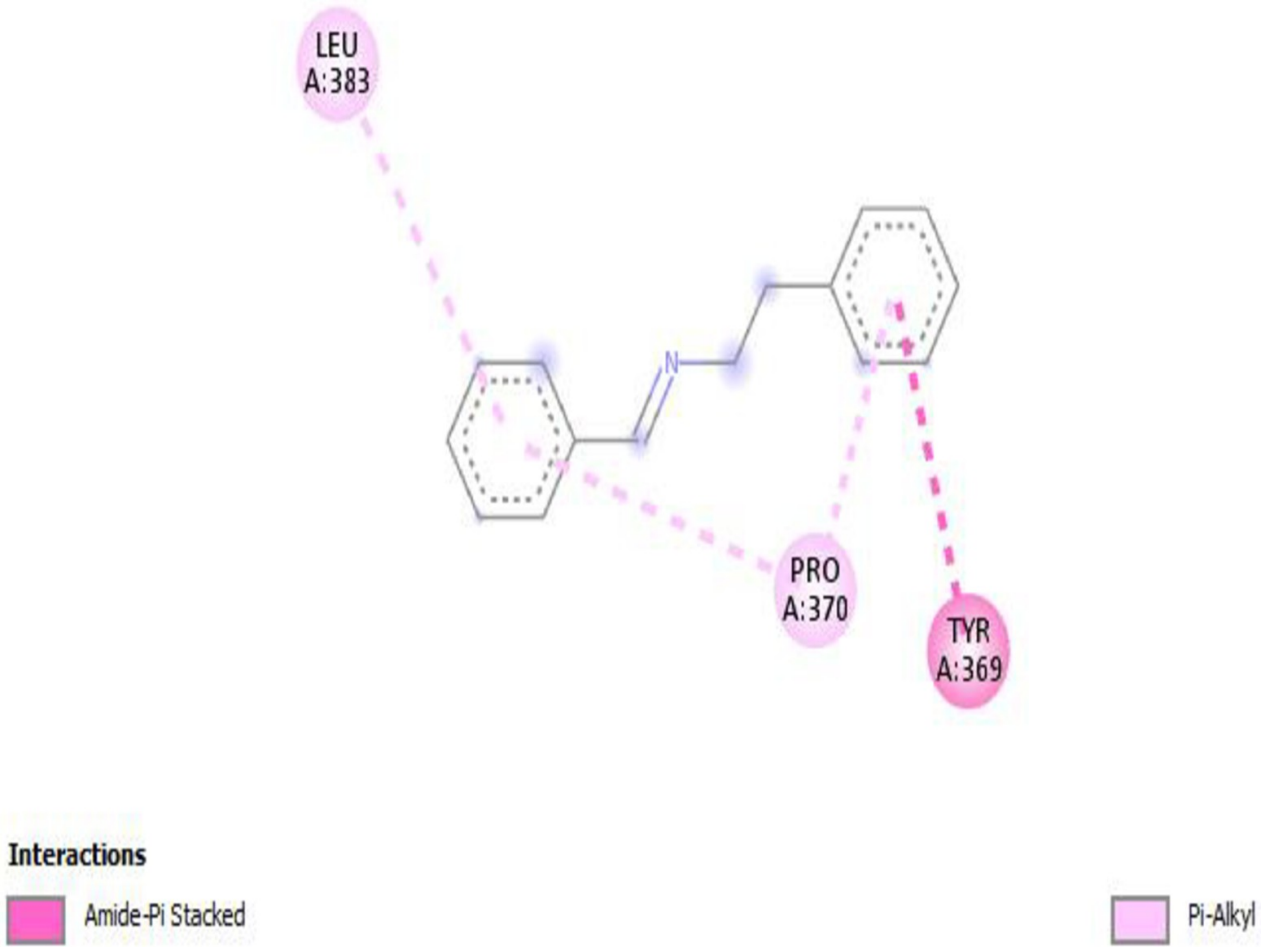
The interaction model of benzeneethanamine ligand with penicillin-binding protein (PBP2a) responsible for the synthesis of the cell wall of *S*. *aureus*.

**Fig 5:**
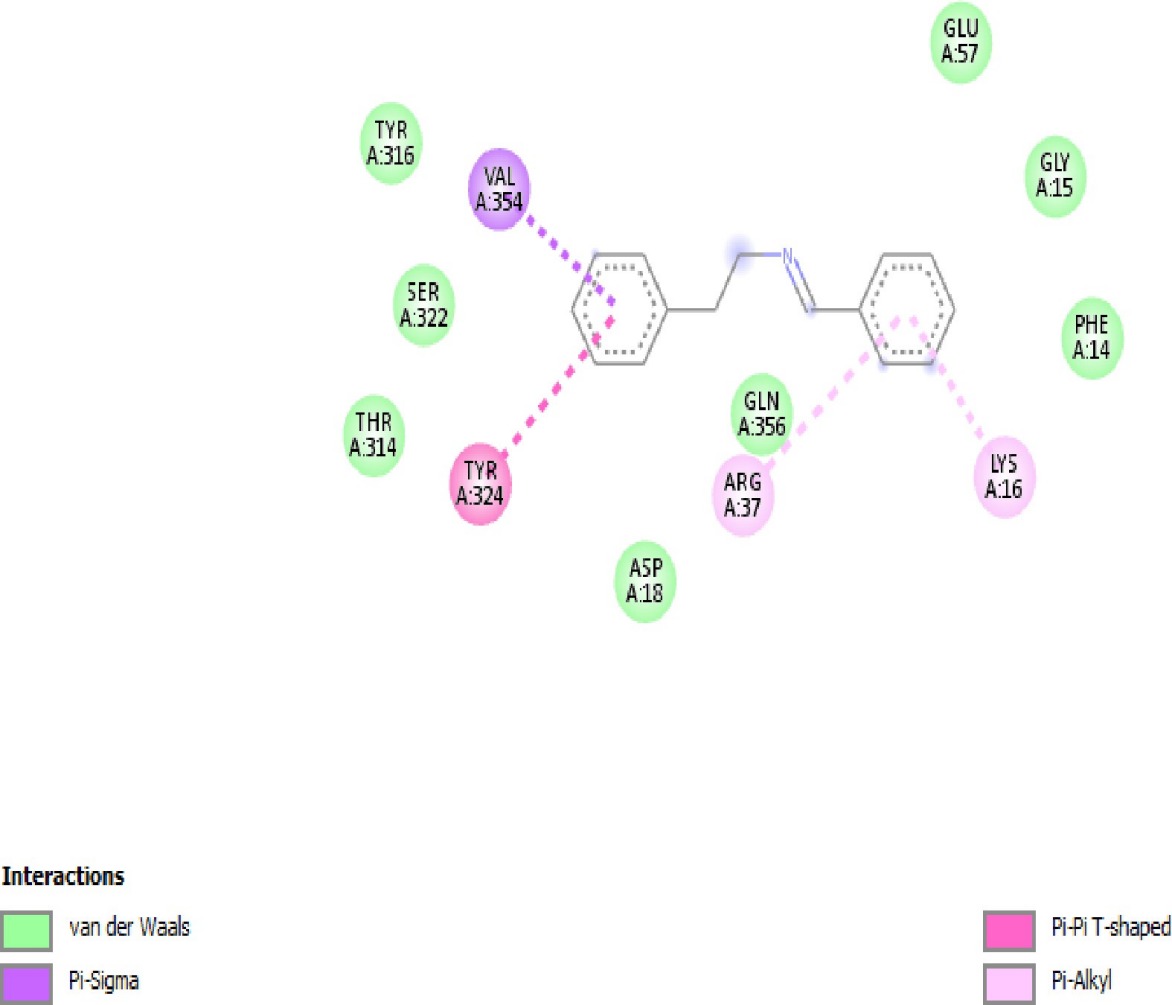
The interaction model of benzeneethanamine ligand with osmoporin (OmpC): An outer membrane protein of *S*. *typhi*.

The identified antibacterial SMs benzeneethanamine with high concentration was selected for molecular docking. The penicillin-binding protein (PBP2a) (PDB ID 5M18) responsible for the synthesis of the cell wall of *S*. *aureus* showed the best interactions with the benzeneethanamine ligand (PubChem CID 576350) with the highest binding energies of −7.2 kcal/mol (**Fig 4**). In *S*. *typhi* strain the osmoporin (OmpC) (PDB ID 3UU2) outer membrane protein which serves as a pore or channel in the outer membrane of the bacterium was selected for the molecular docking. The results showed the best interactions with the benzeneethanamine ligand (PubChem CID 576350) with the highest binding energies of −7.5 kcal/mol (**Fig 5**).

## Discussion

Every year, the MDR pathogenic microorganisms rise as a result of drug abuse [26]. Plant endophytes are known to generate a wide range of metabolites with complex bioactivities in large quantities. Additionally, it is simple and biodegradable to produce these metabolites on a large scale [17, 27]. For the purpose of producing bioactive substances, bacterial endophytes from *K*. *blossfeldiana* were therefore isolated.

In order to eradicate epiphytes on the surface of removed plant tissues, a range of chemical disinfectants have been used. Still, some investigations have demonstrated a notable degree of success when tissue is submerged in ethanol, sodium hypochlorite, and bicarbonate. [17]. In the current study, the same chemical disinfectants were used and exhibited the effectiveness of surface sterilization of the leaves from *K*. *blossfeldiana* which was confirmed as there was no microbial growth observed after 5 days. These suggest that the surface sterility approach was successful in preventing the growth of fungus and bacteria that are epiphytic. As a result, the following isolates are legitimate endophytic bacteria.

Using the agar well diffusion technique, the endophytic *K*. *aerogenes* was screened against two pathogenic human strains, MDR *S*. *aureus* and MDR *S*. *typhi*, to determine its antibacterial efficacy. The tested MDR strains provided MIZ after 24h. The 50μl extract showed activity against *S*. *typhi* while *S*. *aureus* has a moderate inhibition zone. This study is in agreement with [28] where *E*. *coli*, *K*. *pneumonia*, and *S*. *aureus* were the microorganisms against which bacterial endophytes from *Tinospora cordifolia* were evaluated for their possible antibacterial properties through their metabolites.

The previously reported studies show that endophytic *Enterobacter* sp., especially *E*. *cloacae* from banana plants inhibited *Pseudocerocospora fijiensis* which causes black sigatoka disease in bananas (*Musa* sp.). *E*. *cloacae* also efficient plant growth in nutrient-limited soil by N-transfer [29]. In another study, endophytic *E*. *cloacae* isolated from acid lime root also exhibited antagonistic activity against *Phytium aphanidermatum* which causes pythium damping-off disease of cucumber [30]. The present study is in agreement with the previous findings and shows that *Enterobacter* sp. isolated from *K*. *blossfeldiana* successfully inhibits MDR *S*. *typhi* and MDR *S*. *aureus* by the agar well diffusion method.

*Enterobacter* sp., develops metabolites that have antibacterial and antifungal properties and are capable of limiting or suppressing the development of microorganisms e.g. *Candida albicans*, *Bacillus cereus* [31]. Previously, SMs with antimicrobial potentials identified by GC-MS i.e., Benzeneethanamine, Butanoic acid, 3-Hydroxy-2-methylthio-3-phenylpropanoic acid, Thiophene, Propanedinitrile, and 3-Aminopiperidin-2-one etc. developed by *Enterobacter* sp., [30]. In the present study, *K*. *aerogenes* were cultivated and 36 SMs including antibacterial SMs Benzeneethanamine, 3-methyl butanoic acid, 3-Hydroxy-2-methylthio-3-phenylpropanoic acid, 2,5-bis(1,1-dimethylethoxy)-thiophene, 2-bis(3,3-dimethyl-2oxobutylthio) propanedinitrile, 3-aminopiperidin-2-one, 1-ethyl-isoquinoline were identified by GC-MS similar to the previous findings.

The MBC value was determined by observing the least growth of microorganisms upon visual inspection at the lowest concentration of *K*. *aerogenes* extracts during an 18-hour incubation period at 37°C. The MBC value of the extracts in this study was 0.39mg/mL of *S*. *aureus* and *S*. *typhi*. Significant activity against *S*. *typhi* and moderate activity against *S*. *aureus* was demonstrated by the MBC findings in the extracts from *K*. *aerogenes*. These MBC results showed that the extracts exerted moderate antimicrobial activity on the MDR *S*. *aureus* and potentials against *S*. *typhi* strains at different concentrations.

Interestingly, MBC values against the pathogens demonstrate that the extracts are bacteriostatic at lower doses and bactericidal at higher concentrations. These values are greater than MIC. This demonstrates that these *K*. *aerogenes* extracts, when used as antimicrobials, prevent the growth of pathogenic bacteria without necessarily killing them. Since most extract preparations don’t have specified concentrations, this could explain why large amounts of the extracts are used to kill pathogenic bacteria. These observations are in agreement with the research results [32, 33]. The findings of this investigation show that crude extracts with antibacterial properties against pathogenic bacteria are produced by endophytic microorganisms isolated from *K*. *blossfeldiana*. This study confirms the current scientific view that *K*. *blossfeldiana* plant endophytic microbes may be sources of bioactive substances.

The ethyl acetate extracts of endophytic *K*. *aerogenes* showed bacteriostatic properties, according to time-kill kinetic experiments. Several studies have been published on the time-kill kinetics of endophytic bacteria, and several reports on NPs extracts have also been published [34]. The results of the present investigation, however, are not consistent with those of the study [23], which assessed the bactericidal activity of *Trametes gibbosa* extracts by measuring their time-kill kinetic activity. The bioactive antibacterial components in the extracts that are responsible for the antimicrobial action need to be isolated and characterized.

In our study, benzeneethanamine ligand was used for molecular docking studies. The cell wall synthesis protein penicillin-binding protein (PBP2a) for *S*. *aureus* and outer membrane protein osmoporin (OmpC) for *S*. *typhi* was selected. The PBP2a has the highest binding energies of −7.2 kcal/mol while the OmpC exhibits −7.5 binding energies. The present investigation aligns with earlier discoveries [34], demonstrating that the chemicals exhibit affinity and possess a potential lead target towards the broad-spectrum receptor proteins implicated in the manufacture of pathogenic bacterial cell membranes, among which the compound N-(5-benzyl-10b-hydroxy-2-methyl-3,6-dioooctahydro-8H-oxazolo[3,2-α]pyrrolo[2,1c]pyrazin-2-yl)-7-Methyl-2,3,3a,3a1,6,6a,7,8,9,10,10a,10b-dodecahydro-1H-4λ2-indolo[4,3-fg]quinoline-9-carboxamide for instance was ranked as an excellent binder of the MurF enzyme with binding energy −10.2 kcal/mol.

## Conclusions

More research on plant-associated bacterial endophytes has revealed that these organisms have important advantages for a number of industries, including agriculture, industry, and medicines. The results of the present investigation demonstrated that *K*. *blossfeldiana* is indeed composed of bacterial endophytes, which are known to have significant antibacterial properties due to their bioactive components. As a result, we suggest doing additional research to isolate a wider variety of endophytes from *K*. *blossfeldiana*. The potential use of endophytic bacteria is demonstrated by the crude extracts antibiotic capabilities, and as a result, they need to be explored as a novel source for the isolation and synthesis of pure bioactive compounds. In addition, more investigation is required to identify the precise compounds that give rise to the endophytes’ antibacterial properties.

## Supporting information

S1 Fig *K*. *aerogenes* on nutrient agar plate, positive and negative control. (DOCX)

S2 Fig 16S rRNA gene sequences. (DOCX)

S3 Fig GC-MS spectrum of ethyl acetate extracts of *K*. *aerogenes* SMs with peaks of bioactive components. (DOCX)

## References

[1] Ansari S, Nepal HP, Gautam R, Rayamajhi N, Shrestha S, Upadhyay G, et al. Threat of drug-resistant *Staphylococcus aureus* to health in Nepal. BMC Infectious Diseases. 2014; 14:157. doi: 10.1186/1471-2334-14-157 24655316 PMC3994362

[2] Saima Soni I, Lavekar AG, Shukla M, Danish Equbal, Sinha AK, Chopra S. Biocatalytic synthesis of diaryl disulphides and their bio-evaluation as potent inhibitors of drug-resistant *Staphylococcus aureus*. Drug Development Research. 2018;80(1):171–178. doi: 10.1002/ddr.21507 30565263

[3] Kaur G, Balamurugan P, Vasudevan S, Jadav S, Princy SA. Antimicrobial and antibiofilm potential of acyclic amines and diamines against multi-drug resistant *Staphylococcus aureus*. Frontier Microbiology. 2017; 8:1767. doi: 10.3389/fmicb.2017.01767 28966610 PMC5605668

[4] Crump JA, Barrett TJ, Nelson JT, Angulo FJ. Reevaluating fluoroquinolone breakpoints for *Salmonella enterica* serotype *Typhi* and for non-*Typhi salmonellae*. Clinical Infectious Diseases. 2003;37(1):75–81. doi: 10.1086/375602 12830411

[5] Elnekave E, Hong SL, Lim S, Johnson TJ, Perez A, Alvarez J. Comparing serotyping with whole-genome sequencing for subtyping of non-typhoidal *Salmonella enterica*: a large-scale analysis of 37 serotypes with a public health impact in the USA. Microbial Genomics. 2020;6(9): mgen000425. doi: 10.1099/mgen.0.000425 32845830 PMC7643971

[6] Gonzalez-Pimentel JL, Dominguez-Moñino I, Jurado V, Laiz L, Caldeira AT, Saiz-Jimenez C. The rare actinobacterium *Crossiella* sp. is a potential source of new bioactive compounds with activity against bacteria and fungi. Microorganisms. 2022;10(8):1575. doi: 10.3390/microorganisms10081575 36013993 PMC9415966

[7] Girão M, Ribeiro I, Ribeiro T, Azevedo IC, Pereira F, Urbatzka R, et al. *Actinobacteria* isolated from *Laminaria ochroleuca*: a source of new bioactive compounds. Frontier Microbiology. 2019;10. doi: 10.3389/fmicb.2019.00683 31024480 PMC6465344

[8] Gulsen SH, Tileklioglu E, Bode E, Cimen H, Ertabaklar H, Ulug D, et al. Antiprotozoal activity of different Xenorhabdus and Photorhabdus bacterial secondary metabolites and identification of bioactive compounds using the easy PACId approach. Scientific Reports. 2022;12(1). doi: 10.1038/s41598-022-13722-z 35750682 PMC9232601

[9] Mohan CD, Rangappa S, Nayak SC, Jadimurthy R, Wang L, Sethi G, et al. Bacteria as a treasure house of secondary metabolites with anticancer potential. Cancer Biology. 2022;86(Pt 2):998–1013. doi: 10.1016/j.semcancer.2021.05.006 33979675

[10] Isolation El-Deeb B. and characterization of endophytic bacteria from *Plectranthus tenuiflorus* medicinal plant in Saudi Arabia desert and their antimicrobial activities. Journal of Plant Interactions. 8:1, 56–64, doi: 10.1080/17429145.2012.680077

[11] Wang S, Liu J, Sun J, Sun Y, Liu J, Jia N, Ostrikov K. Diversity of culture-independent bacteria and antimicrobial activity of culturable endophytic bacteria isolated from different Dendrobium stems. Scientific Report. 2019; (17)9. doi: 10.1038/s41598-019-46863-9 31316117 PMC6637234

[12] Gouda S, Das G, Sen SK, Shin HS, Patra JK. Endophytes: a treasure house of bioactive compounds of medicinal importance. Frontier Microbiology. 2016; 7:1538. doi: 10.3389/fmicb.2016.01538 27746767 PMC5041141

[13] Monowar T, Rahman MS, Bhore SJ, Raju G, Sathasivam KV. Silver nanoparticles synthesized by using the endophytic bacterium *Pantoea ananatis* are promising antimicrobial agents against multidrug-resistant bacteria. Molecules. 2018;23(12):3220. doi: 10.3390/molecules23123220 30563220 PMC6321088

[14] Costa SS, Muzitano MF, Camargo LM, Coutinho MA. Therapeutic potential of *Kalanchoe* species: flavonoids and other secondary metabolites. Natural Product Communications. 2008: 3 doi: 10.1177/1934578X0800301236

[15] Ding T, Melcher U. Influences of plant species, season and location on leaf endophytic bacterial communities of non-cultivated plants. PloS one. 2016;11(3): doi: 10.1371/journal.pone.0150895 26974817 PMC4790846

[16] Maela MP, van der Walt H, Serepa-Dlamini MH. The antibacterial, antitumor activities, and bioactive constituents’ identification of *Alectra sessiliflora* bacterial endophytes. Frontiers in Microbiology. 2022; 13:870821. doi: 10.3389/fmicb.2022.870821 35865925 PMC9294510

[17] Nxumalo CI, Ngidi LS, Shandu JS, Maliehe TS. Isolation of endophytic bacteria from the leaves of *Anredera cordifolia* CIX1 for metabolites and their biological activities. BMC Complementary Medicine and Therapies. 2020;20(1):1–1, 300. doi: 10.1186/s12906-020-03095-z 33028279 PMC7541265

[18] Kim HY, Choi GJ, Lee HB, Lee SW, Lim HK, Jang KS, et al. Some fungal endophytes from vegetable crops and their anti-oomycete activities against tomato late blight. Letters in Applied Microbiology. 2007;44(3):332–337. doi: 10.1111/j.1472-765X.2006.02093.x 17309513

[19] Photolo MM, Mavumengwana V, Sitole L, Tlou MG. Antimicrobial and antioxidant properties of a bacterial endophyte, *Methylobacterium radiotolerans* MAMP 4754, isolated from *Combretum erythrophyllum* seeds. International Journal of Microbiology. 2020; 2020:9483670. doi: 10.1155/2020/9483670 32184829 PMC7060864

[20] Feng Y, Zhang Y, Shah OU. Isolation and Identification of Endophytic Bacteria *Bacillus* sp. ME9 That Exhibits Biocontrol Activity against *Xanthomonas phaseoli* pv. *manihotis*. Biology; 12. 2023. doi: 10.3390/biology12091231 37759630 PMC10525512

[21] Mira P, Yeh P, Hall BG. Estimating microbial population data from optical density. PloS one; 17. Epub ahead of print October 13, 2022. doi: 10.1371/journal.pone.0276040 36228033 PMC9562214

[22] Olajuyigbe OO, Afolayan AJ. In vitro antibacterial and time-kill evaluation of the *Erythrina caffra thunb*. extract against bacteria associated with diarrhea. Scientific World Journal. 2012;738314. doi: 10.1100/2012/738314 23213297 PMC3504411

[23] Appiah T, Boakye YD, Agyare C. Antimicrobial activities and time-kill kinetics of extracts of selected Ghanaian mushrooms. Evid Based Complement Alternative Medicines. 2017; 2017:4534350. doi: 10.1155/2017/4534350 29234399 PMC5682094

[24] Meng XY, Zhang HX, Mezei M, Cui M. Molecular docking: a powerful approach for structure-based drug discovery. Current Computer Aided Drug Des. 2011;7(2):146–157. doi: 10.2174/157340911795677602 21534921 PMC3151162

[25] Ullah S, Muhammad Mohsin Raza, Abbas T, Guan X, Zhou, He P. Responses of soil microbial communities and enzyme activities under nitrogen addition in fluvo-aquic and black soil of North China. Frontier Microbiology. 2023;14. doi: 10.3389/fmicb.2023.1249471 37664123 PMC10469899

[26] Mancuso G, Midiri A, Gerace E, Biondo C. Bacterial antibiotic resistance: the most critical pathogens. Pathogens. 2021;10(10):1310. doi: 10.3390/pathogens10101310 34684258 PMC8541462

[27] Pereira-Dias L, Oliveira-Pinto PR, Fernandes JO, Regalado L, Mendes R, Teixeira C, et al. Peptaibiotics: harnessing the potential of microbial secondary metabolites for mitigation of plant pathogens. Biotechnology Advances. 2023; 68:108223. doi: 10.1016/j.biotechadv.2023.108223 37536466

[28] Macedo-Raygoza GM, Valdez-Salas B, Prado FM, Prieto KR, Yamaguchi LF, Kato MJ, et al. *Enterobacter cloacae*, an endophyte that establishes a nutrient-transfer symbiosis with banana plants and protects against the black *Sigatoka* pathogen. Frontier Microbiology. 2019; 10:804. doi: 10.3389/fmicb.2019.00804 31133991 PMC6513882

[29] Kazerooni EA, Al-Shibli H, Nasehi A, Al-Sadi AM. Endophytic *Enterobacter cloacae* exhibits antagonistic activity against Pythium damping-off of cucumber. Ciencia Rural. 2010;50(8):1–7. doi: 10.1590/0103-8478cr20191035

[30] Hameed RH, Abbas FM, Hameed IH. Bioactive chemical analysis of *Enterobacter aerogenes* and test of its antifungal and antibacterial activity and determination. Indian Journal Public Health Res Dev. 2018;9(5):442–448.

[31] Monowar T, Rahman MS, Bhore SJ, Sathasivam KV. Endophytic Bacteria *Enterobacter hormaechei* Fabricated Silver Nanoparticles and Their Antimicrobial Activity. Pharmaceutics. 2021;13(4):511. doi: 10.3390/pharmaceutics13040511 33917798 PMC8068190

[32] Avinash KS, Ashwini HS, Babu HNR, Krishnamurthy YL. Antimicrobial potential of crude extract of *Curvularia lunata*, an endophytic fungi isolated from *Cymbopogon caesius*. Journal of Mycology. 2015; 2015:185821. doi: 10.1155/2015/185821

[33] Shen N, Chen Z, Cheng G, Lin W, Qin Y, Xiao Y. Diversity, chemical constituents and biological activities of endophytic fungi from *Alisma orientale* (Sam.) Juzep. Frontiers in Microbiology. 2023; (14)6. doi: 10.3389/fmicb.2023.1190624 PMC10320293 37415810

[34] Rahman L, Mukhtar A, Ahmad S, Ali M, Saeed M, Shinwari ZK. Endophytic bacteria of *Fagonia indica Burm*. f revealed to harbor rich secondary antibacterial metabolites. PloS one. 2022;17(12): e0277825. doi: 10.1371/journal.pone.0277825 36520861 PMC9754247

