# Expression of Concern: Exploration of *Klebsiella aerogenes* derived secondary metabolites and their antibacterial activities against multidrug-resistant bacteria

**DOI:** 10.1371/journal.pone.0357416

**Published:** 2026-09-02

**Authors:** The PLOS One Editors

The *PLOS One* Editors issue an Expression of Concern for this article [1] to notify readers of concerns about the article’s compliance with the PLOS Authorship policy.

In light of the concerns, the *PLOS One* Editors issue this Expression of Concern. Readers are advised to interpret the article [1] with caution.
