# Supplementary figures and images for: Exploration of Klebsiella aerogenes derived secondary metabolites and their antibacterial activities against multidrug-resistant bacteria
Source: PLoS One. 2024 Sep 16;19(9):e0300979. doi: 10.1371/journal.pone.0300979 (PMC11404795; doi:10.1371/journal.pone.0300979)

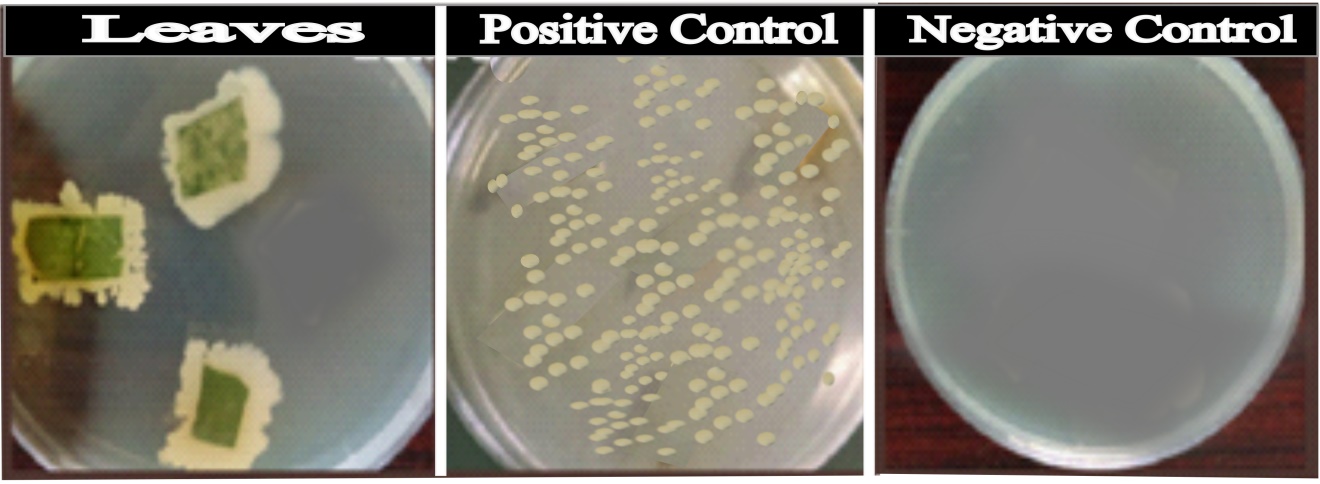


**S_Fig 1.** K. aerogene on nutrient agar plate, positive and negative control

Supplement: S1 Fig — (DOCX) [file pone.0300979.s001.docx]

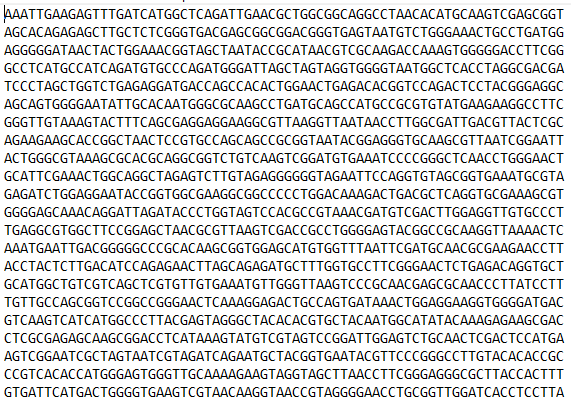


**S_Fig 2.** 16S rRNA gene sequences

Supplement: S2 Fig — (DOCX) [file pone.0300979.s002.docx]
