# Supplementary material for: Exploration of Klebsiella aerogenes derived secondary metabolites and their antibacterial activities against multidrug-resistant bacteria
Source: PLoS One. 2024 Sep 16;19(9):e0300979. doi: 10.1371/journal.pone.0300979 (PMC11404795; doi:10.1371/journal.pone.0300979)

**S_Fig 3.** GC-MS spectrum of ethyl acetate extracts of *K. aerogene* SMs with peaks of bioactive components
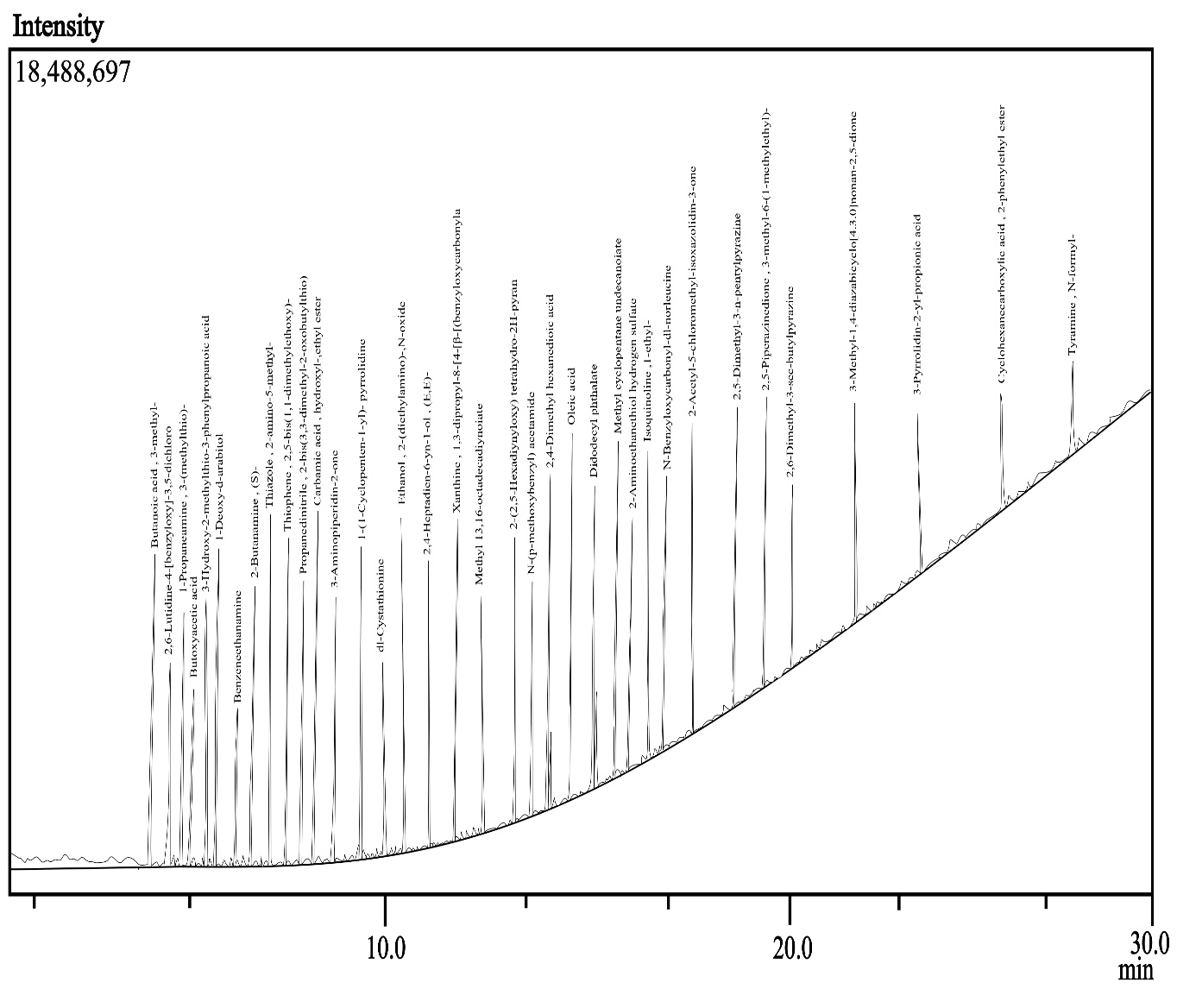

Supplement: S3 Fig — (DOCX) [file pone.0300979.s003.docx]
